# Traditional Uses of *Thymus* Species in the Balkans: Ethnopharmacology, Food, and Cultural Heritage

**DOI:** 10.3390/life16030452

**Published:** 2026-03-10

**Authors:** Ina Aneva, Marija Marković, Katarina Husnjak Malovec, Zheko Naychov, Irena Mincheva, Vesna Stankov-Jovanović, Ekaterina Kozuharova

**Affiliations:** 1 Bulgarian Academy of Sciences, 1000 Sofia, Bulgaria; 2 Institute of Forestry, 11030 Belgrade, Serbia; markovicsmarija9@gmail.com; 3 Faculty of Science, University of Zagreb, 10000 Zagreb, Croatia; katarina.husnjak.malovec@biol.pmf.hr; 4 University Hospital Lozenets, Department of Surgery, Sofia University St. Kliment Ohridski, 1504 Sofia, Bulgaria; zh.naychov@gmail.com; 5 Institute of Chemical Engineering, Bulgarian Academy of Sciences, 1113 Sofia, Bulgaria; irenamincheva@gmail.com; 6 Faculty of Sciences and Mathematics, University of Niš, 18000 Niš, Serbia; vesna.stankov-jovanovic@pmf.edu.rs; 7 Faculty of Pharmacy, Department of Pharmacognosy, Medical University, 1000 Sofia, Bulgaria

**Keywords:** *Thymus*, Balkan Peninsula, ethnopharmacology, traditional medicine, food uses, medicinal plants, cultural traditions, taxonomic issues

## Abstract

*Thymus* species play an important role in traditional medicine, food practices, and cultural heritage across the Balkan Peninsula, a region characterized by high floristic diversity and long-standing ethnobotanical traditions. This review provides a comprehensive synthesis of published ethnobotanical and ethnopharmacological data on the traditional uses of *Thymus* species in the Balkans. A systematic survey of literature published between 1900 and 2022 was conducted using major scientific databases and classical ethnobotanical sources, covering Albania, Bosnia and Herzegovina, Bulgaria, Croatia, Greece, Kosovo, Montenegro, North Macedonia, Romania, Serbia, Slovenia, and the European part of Turkey. In total, 553 records of traditional use were documented. The results reveal that *Thymus* taxa are most frequently employed for the treatment of respiratory and gastrointestinal disorders, followed by applications related to the nervous, urinary, cardiovascular, and integumentary systems. Culinary uses such as spices, herbal teas, beverages, and food preservatives are widely reported, highlighting the close connection between medicinal and dietary traditions. The analysis also identifies significant taxonomic inconsistencies in the ethnobotanical literature, particularly the frequent misapplication of names such as *T. serpyllum* and *T. vulgaris*, which complicates the interpretation of traditional knowledge. Overall, the strong cross-cultural consistency of uses across Balkan countries supports the pharmacological relevance of *Thymus* species and aligns well with modern phytotherapeutic evaluations. This review underscores the importance of critically integrating ethnobotanical data, taxonomic accuracy, and contemporary phytotherapy to better understand and utilize the therapeutic potential of *Thymus* species.

## 1. Introduction

Over the centuries, thyme has been associated with a broad spectrum of medicinal effects, many of which were initially documented through empirical observation and folk tradition. Since ancient times, these plants have been used for treating respiratory disorders, digestive ailments, and common colds [1]. They are known for a broad spectrum of biological properties, including expectorant, antiseptic, antifungal, spasmolytic, carminative, sedative, diaphoretic, and diuretic effects [2,3,4,5]. These activities, however, may vary considerably among species, reflecting differences in chemical composition. Beyond its well-documented biological activities, the genus *Thymus* presents significant scientific challenges related to its taxonomic complexity. The genus comprises more than 250 perennial herbaceous or semi-shrubby species distributed in eight sections [6], while Flora Europaea lists 66 species with numerous subspecies and varieties [7]. Difficulties in species identification have limited the number of phytochemical studies conducted at the species level. In industrial practice, different taxa are frequently collected and marketed under the general name “*Thymus* sp.” (thyme), which overlooks chemical specificity and complicates both pharmacological standardization and the interpretation of ethnobotanical data.

Traditional uses of *Thymus* species encompass both external and internal applications, reflecting their multifunctional role in folk medicine. Bardeau [8] considered thyme indispensable for maintaining health, describing its invigorating and mood-lifting qualities when consumed as an infusion. Ethnopharmacological records describe topical applications of thyme-based decoctions and essential oils for managing wounds, ulcers, skin infections, abscesses, and various forms of dermatitis [9,10]. Its use in massages to relieve rheumatic pain, sciatica, arthritis, and neuralgia has also been recorded [11]. Additionally, thyme baths have been regarded as both stimulating and relaxing, sometimes recommended as part of slimming therapies [12,13].

Among the various traditional applications, internal uses of *Thymus* species are particularly prominent. In this context, respiratory health represents the main therapeutic focus. Due to its expectorant, spasmolytic, and antiseptic activities, thyme preparations are widely used for treating bronchitis, cough, asthma, sinusitis, and even tuberculosis [8,10,14,15]. Moreover, thyme is valued as a general tonic, reportedly alleviating insomnia, nervous tension, anxiety, and fatigue [8,14]. Its application extends to gastrointestinal disorders, including dyspepsia, colic, diarrhea, flatulence, and gastric ulcers, as well as parasitic infections [9,16,17]. In traditional medicine, its diuretic and antiseptic properties have also been used for genitourinary conditions, and cardiovascular benefits such as improved circulation and cholesterol regulation have been reported [18].

The Balkan Peninsula, characterized by high floristic diversity, complex geological structure, and heterogeneous environmental conditions, holds a long-standing tradition of utilizing *Thymus* species in both culinary and medicinal contexts [19,20]. The region represents a biocultural hotspot where high plant diversity intersects with deeply rooted ethnobotanical traditions. Local communities have relied on thyme infusions, decoctions, and ointments for respiratory ailments, digestive issues, and general wellness. The region is home to numerous *Thymus* taxa, several of which are endemic, and their traditional use represents an important link between biodiversity and cultural heritage.

From a modern phytotherapeutic perspective, such traditional knowledge is complemented by standardized quality controls and clinical evaluation. In Europe, the German Commission E monographs, first published in 1978, formally assessed the safety and efficacy of over 300 medicinal plants, including *Thymus* [21]. Similar efforts by the European Scientific Cooperative on Phytotherapy [22] and the World Health Organization [23] have reinforced the medicinal relevance of thyme by establishing pharmacological guidelines and quality standards. These evaluations highlight the importance of thyme among herbal remedies and ensure its continued use in both traditional and modern healthcare.

Given this historical depth and ethnobotanical significance, the present ethnobotanical study focuses on the traditional use of *Thymus* species on the Balkan Peninsula, aiming to document local practices and evaluate their relevance to contemporary phytotherapy. In particular, it seeks to synthesize ethnobotanical records across multiple Balkan countries, compare patterns of traditional use, and highlight taxonomic ambiguities affecting the interpretation of these data.

## 2. Materials and Methods

For the purposes of this study, the Balkan Peninsula is considered to include Albania, Bosnia and Herzegovina, Bulgaria, Croatia, Kosovo, Montenegro, North Macedonia, Romania, Serbia, and Slovenia, with all or part of each country lying within the peninsula; however, there is no universal consensus regarding the exact boundaries of the region [24]. The present work represents a qualitative ethnobotanical synthesis integrating heterogeneous historical and contemporary sources rather than a strictly structured systematic review or meta-analysis.

### 2.1. Data Search Engines and Procedures

To identify relevant literature published between 1900 and 2022, we consulted classical ethnobotanical books and conducted systematic searches in Google Scholar, Web of Science, and PubMed. Searches were performed using combinations of country names (“Albania”, “Bosnia and Herzegovina”, “Bulgaria”, “Croatia”, “Greece”, “Kosovo”, “Montenegro”, “North Macedonia”, “Romania”, “Slovenia”, “Serbia”, and the European part of “Turkey”) and keywords such as “*Thymus*”, “thyme”, “traditional”, “medicinal”, “food”, “ethnobotany”, and “ethnopharmacology”.

Following the PRISMA 2020 guidelines [25], all records were screened for eligibility. The literature search and screening were conducted in a decentralized manner by co-authors from different Balkan countries, each independently consulting national-language sources, regional ethnobotanical literature, and international databases relevant to their geographic area of expertise. The collected records were subsequently consolidated and harmonized for qualitative synthesis. PRISMA 2020 recommendations were applied as a general framework to enhance transparency in reporting eligibility and exclusion criteria; however, a centralized reference library with unified record tracking was not established at the initial stage of the study, and therefore a formal PRISMA flow diagram with precise counts could not be reconstructed retrospectively. During the consolidation process, duplicate records retrieved from multiple databases were identified and removed. Overlapping ethnobotanical reports were critically evaluated to avoid artificial inflation of use records, and similar traditional uses were considered independent only when they originated from distinct field studies, geographical regions, or informant groups. The screening process included evaluation of titles, abstracts, and full texts. A total of 87 publications were excluded for the following reasons: (i) the information was not relevant to the research topic; (ii) the data pertained exclusively to medicinal uses of plants without ethnobotanical context; (iii) the studies addressed traditional food practices but did not include wild plant species; and (iv) the records referred solely to the consumption of cultivated plants.

From the selected publications, we extracted information on traditionally used *Thymus* taxa (reported as *Thymus* sp. div.) for medicinal and food purposes in Albania, Bosnia and Herzegovina, Bulgaria, Croatia, Greece, Kosovo, Montenegro, North Macedonia, Romania, Slovenia, Serbia, and the European part of Turkey.

### 2.2. Data Set Preparation and Analyses

**Figure 1:**
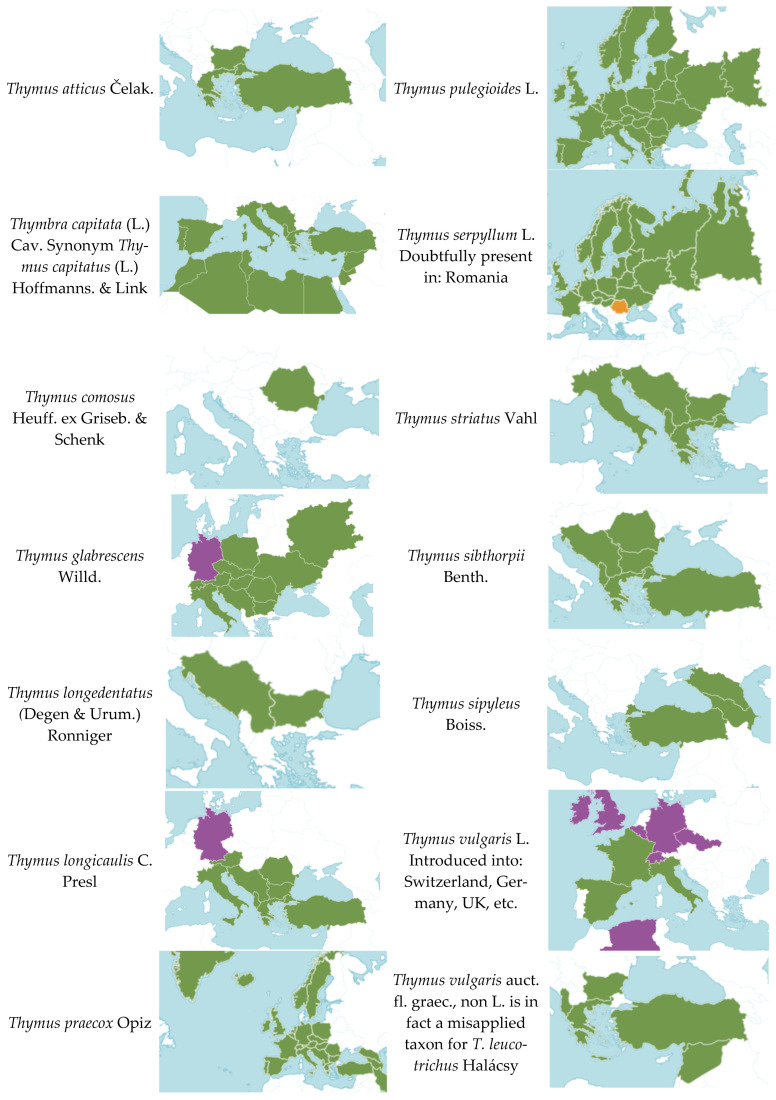
Taxa reported by ethnobotanical studies across Balkan countries. Legend green color—native, purple color—introduced, orange color—doubtfully present.

The reported data for each country were organized in Excel tables and analyzed according to categories of medicinal application. The taxonomic treatment of the reported taxa follows the original sources; subsequent clarification and verification of accepted names and distribution were conducted in accordance with POWO [26] (Figure 1).

## 3. Results and Discussion

The ethnobotanical and ethnopharmacological survey of *Thymus* species across the Balkan countries reveals a rich and diverse spectrum of traditional uses, with notable consistency in medicinal applications despite regional differences. In addition to medicinal purposes, thyme is widely used as a culinary seasoning and in traditional treatments of livestock. Data collected from Albania, Bulgaria, Bosnia and Herzegovina, Croatia, Greece, Kosovo, Montenegro, North Macedonia, Serbia, Slovenia, southern Romania, and the European part of Turkey indicate that thyme is primarily used for the treatment of respiratory and gastrointestinal disorders, followed by dermatological, musculoskeletal, and other conditions (Table 1, Table 2 and Table S1). Several limitations were identified, including uneven geographical coverage of ethnobotanical studies across the Balkan countries and the impossibility of precise identification for some reported taxa.

### 3.1. Regional Diversity of Species

This section presents an integrated overview combining descriptive ethnobotanical data with interpretative taxonomic discussion in order to reflect the close relationship between reported uses and species identification. The Balkan Peninsula is home to numerous *Thymus* species, several of which are endemic, exhibiting notable differences in their chemical profiles and, consequently, biological activities. For example, the essential oils of *T. sibthorpii*, *T. pulegioides*, *T. glabrescens*, and *T. callieri* are rich in linalool (22–51%), whereas this compound is virtually absent in *T. zygioides*. Thymol is absent in the essential oils of these species except for *T. glabrescens*, where it reaches up to 35%, while geraniol content varies between 0 and 28% among these taxa [27].

The predominance of respiratory indications across the Balkan Peninsula is consistent with the widespread occurrence of thymol- and carvacrol-rich chemotypes, both compounds being well known for their antimicrobial, expectorant, and spasmolytic activities. In contrast, taxa characterized by higher linalool content may partly explain the reported sedative and neurorelaxant uses in certain regions. This chemotypic variability provides a plausible phytochemical basis for the diversity of traditional applications recorded in the ethnobotanical literature.

We summarized the data on *Thymus* species used in each country and their reported applications for various health conditions, as presented in the original sources (Table 2 and Table S1). As a result, fourteen taxa belonging to the genus *Thymus* were identified (Figure 1). This pattern reflects widespread name misapplication in ethnobotanical sources and highlights persistent taxonomic ambiguities within the genus. However, *Thymus serpyllum* is not distributed in the Balkan Peninsula (Figure 1; [26]). Nevertheless, this species is officially recognized as Serpylli herba in the European Pharmacopoeia [28], which likely explains the frequent use of this name in anecdotal and ethnobotanical reports. Similarly, *Thymus vulgaris* does not occur in the Balkan Peninsula (Figure 1; [26]). This likely explains the frequent use of this name in anecdotal reports, as species within the genus *Thymus* are often difficult to distinguish morphologically, particularly in field-based ethnobotanical surveys. Therefore, such mentions should be interpreted with caution and treated as *Thymus* sp. div. In the tables and summaries, species names are retained exactly as reported in the original ethnobotanical sources to ensure traceability of the published records.

The taxon often referred to as the Mediterranean thyme, *Thymus vulgaris* auct. fl. graec., non L., represents a misapplied name that actually corresponds to *Thymus leucotrichus* Halácsy [29,30]. Furthermore, it is not possible to determine which species is denoted by the name *Thymus* aff. *comosus* Heuffel ex Griseb. reported by Šarić Kundalić et al. [31], as *Thymus* comosus does not occur in the Balkan Peninsula (Figure 1).

In contrast to these misapplied or ambiguous names, localized species such as *Thymus longedentatus* in Bulgaria and *Thymus atticus* in Greece hold significant ethnomedicinal value. This taxonomic and cultural diversity not only enriches the regional heritage but also offers important opportunities for further phytochemical and pharmacological research.

It is worth noting that, according to the Flora of Serbia, *Thymus marschallianus* Willd. is treated as an accepted species, whereas the POWO and Euro+Med checklists consider this name to be a synonym of *Thymus pannonicus*.

Although *Thymus vulgaris* L. does not naturally occur in the Balkan Peninsula according to the POWO and Euro + Med checklists, it is widely cultivated in several Balkan countries [32].

### 3.2. Ethnopharmacological Data

Given the heterogeneous nature of ethnobotanical sources, descriptive results and interpretative analysis are presented together to facilitate cross-cultural comparison and contextual understanding.

#### 3.2.1. Health Conditions Treated with Thyme and Other Applications

A total of 553 records of traditional medicinal use of thyme for various health conditions were documented across the Balkan countries (Table 1). The number of records varies considerably among countries, with particularly high representation in Serbia and Bosnia and Herzegovina, and comparatively few records reported from Montenegro and Slovenia. Most records refer to the use of *Thymus* species for the treatment of respiratory disorders, followed by gastrointestinal conditions (Table 1).

Ten taxa of *Thymus* (including representatives of *Thymbra*), namely *T. aff. comosus*, *T. capitatus*, *T. glabrescens*, *T. longedentatus*, *T. longicaulis*, *T. praecox*, *T. pulegioides*, *T. serpyllum*, *T. sipyleus*, and *T. vulgaris*, as well as unspecified records reported as *Thymus* sp. div., are traditionally used in Balkan countries for the treatment of respiratory disorders. These include conditions such as sore throat and throat inflammation, pharyngitis, angina, asthma, bronchial spasms, bronchitis, chills, common cold, dry, productive, whooping, and regular cough, diphtheria, fever, influenza, lung inflammation, and other respiratory infections and inflammations (Table 2 and Table S1, Supplementary Material). Reported activities include antitussive, expectorant, antipyretic, and mucolytic effects (Table 1, Table 2 and Table S1, Supplementary Material). For respiratory conditions, thyme is most commonly administered as an infusion, although inhalation is also frequently reported.

The second most frequently reported therapeutic use involves the treatment of gastrointestinal disorders, such as diarrhea and stomachache, with preparations usually administered as infusions. Records for this application involve twelve *Thymus* taxa, namely *T. atticus*, *T. aff. comosus T. capitatus*, *T. glabrescens*, *T. longedentatus*, *T. longicaulis*, *T. praecox*, *T. pulegioides*, *T. sibthorpii*, *T. sipyleus*, *T. vulgaris*, *T. zygioides* as well as unspecified *T.* sp. div. including *T. serpyllum* (Table 1, Table 2 and Table S1/Supplementary Material).

The third most frequently reported therapeutic use concerns disorders of the nervous system, including anxiety, convulsions, dizziness, epilepsy, eye inflammations, headache, hand tremor, hysteria, insomnia, neurasthenia, neurosis, psychotic states, tremor, nervous tension, and conditions described in folk terminology as “for nerves”, “feeling anxious/tense”, or “nervous troubles”, including nervousness related to alcoholism (Table 1, Table 2 and Table S1, Supplementary Material). In most cases, plant material is administered as an infusion; however, insomnia is also treated using pillow fillings or whole-body baths, particularly in cases of neurasthenia (Table 1, Table 2 and Table S1, Supplementary Material). Taxa reported for the treatment of nervous system disorders include *T. aff. comosus*, *T. longedentatus*, *T. praecox*, *T. pulegioides*, *T. vulgaris*, and *T. zygioides*, as well as records reported as *Thymus* sp. div. and *T. serpyllum* (Table 1, Table 2 and Table S1, Supplementary Material).

Disorders of the urinary system are treated with infusions prepared from *T. aff. comosus*, *T. longedentatus*, *T. longicaulis*, *T. praecox*, *T. pulegioides*, *T. serpyllum*, *T. sibthorpii*, *T. vulgaris*, and unspecified *Thymus* sp. div. Thyme is also reported to alleviate more complex conditions, such as kidney stones and nephritis (Table 1, Table 2 and Table S1, Supplementary Material).

Nine *Thymus* taxa, namely *T. aff. comosus*, *T. capitatus*, *T. glabrescens*, *T. longedentatus*, *T. longicaulis*, *T. praecox*, *T. pulegioides*, *T. sipyleus*, and *T. vulgaris*, as well as unspecified records reported as *Thymus* sp. div. and *T. serpyllum*, are traditionally used in Balkan countries for the treatment of cardiovascular conditions. Reported applications include anemia, “blood purification”, heart-related disorders, high blood pressure/hypertension, improvement of blood circulation, and strengthening of the heart muscle (Table 2 and Table S1, Supplementary Material).

Six *Thymus* taxa, namely *T. aff. comosus*, *T. glabrescens*, *T. longedentatus*, *T. praecox*, *T. pulegioides*, and *T. vulgaris*, as well as unspecified records reported as *Thymus* sp. div. and T. serpyllum, are traditionally used to alleviate disorders related to the immune system. These applications include strengthening of the body, disease prevention, immunostimulation, use as roborants, and treatment of lymph node inflammation. Preventive use against infectious diseases is also frequently reported (Table 2 and Table S1, Supplementary Material).

Skin-related conditions are treated with nine *Thymus* taxa, as well as with unspecified *Thymus* sp. div. and *T. serpyllum*, whereas fewer taxa are reported for the treatment of metabolic disorders (Table 2 and Table S1, Supplementary Material). Due to the high diversity of reported traditional uses across countries and taxa, a detailed tabular presentation is provided to preserve the full ethnobotanical context and ensure transparency of the compiled data.

Limitations of traditional medicine are related to the fact that treatments are generally based on observable symptoms rather than on underlying causes. From the perspective of contemporary institutional medicine, similar symptoms may originate from different physiological processes and therefore require distinct therapeutic approaches. For instance, folk healers did not clearly distinguish cardiac from gastric ailments; conditions described as “heart pain” were most likely what is now understood as stomachache. In other cases, corresponding modern medical categories simply did not exist. Because blood pressure could not be measured using traditional techniques, healers had no basis for identifying conditions such as arterial hypertension. Consequently, traditional “diagnoses” represent observations of symptoms rather than formal disease classifications. A similar interpretation applies to treatments described as “blood purification”.

Nonetheless, systematic scholarly investigation and critical interpretation of these practices can reveal valuable information about the pharmacological potential of thyme.

Culinary applications of thyme are widely reported across the Balkan Peninsula. These include use as a food seasoning, particularly in dried and powdered form [33,34]; preparation of beverages, such as teas or cold-water macerations of fresh herbs with sugar; and use as a spice for meat dishes in Bulgaria [35,36]. In North Macedonia, fresh thyme is commonly used as a spice in salads and meat dishes [37]. In Slovenia, thyme is used as a seasoning for soups, meat, eggs, fruit and vegetable dishes, desserts, beverages, and schnapps [38]. In Serbia, it is used both as a culinary spice and for washing glassware and protecting pickled vegetables [39]). In Croatia, thyme is employed as a seasoning and in the preparation of food and liqueurs [40,41]. In Romania, it is traditionally used as a seasoning for homemade “borsch” [42], while in Turkey it is commonly used as a spice [43].

Beyond medicinal and culinary uses, dried flowering branches of thyme are burned during religious ceremonies in Bosnia and Herzegovina [44], particularly during funerals, and similar practices have been documented in Bulgaria [45].

#### 3.2.2. Albania

Traditional medicinal use of thyme is documented 37 times across nine publications (Table 1, Table 2 and Table S1), including studies by Rexhepi et al. [46,47], Pieroni et al. [33,34,48], Barina et al. [49], Tomasini et al. [50], Aziz et al. [51], and Icka et al. [52]. *Thymus* species are predominantly used for the treatment of disorders affecting the respiratory, nervous, and gastrointestinal systems (Table 1). *T. pulegioides* is specifically reported as a remedy for headache in Albania [34,52]. *T. longicaulis* is used to treat a wide range of ailments and is even described in some sources as a panacea [48,51,52]. In both Albania and Kosovo, *Thymus serpyllum* is among the most frequently used taxa for the preparation of teas and infusions. It is applied to treat respiratory inflammations, bronchitis, asthma, gastrointestinal disorders, and influenza, as well as to improve blood circulation, and is also reported as an immunostimulant, neurorelaxant, carminative, spasmolytic, and sedative agent [52]. In both regions, *T. vulgaris* L. is reported as a popular antitussive and anticholesterolemic tea infusion [52]. Additionally, *T. pulegioides* is among approximately 30 medicinal plant species that represent an important source of income for many families in the Mount Korab villages (Bellovë, Rabdisht, Cerjan, and Zagrad) in the Peshkopia area of eastern Albania. In this context, the species is also used as a food seasoning, primarily in dried and powdered form [33].

#### 3.2.3. Bosnia and Herzegovina

**Figure 2:**
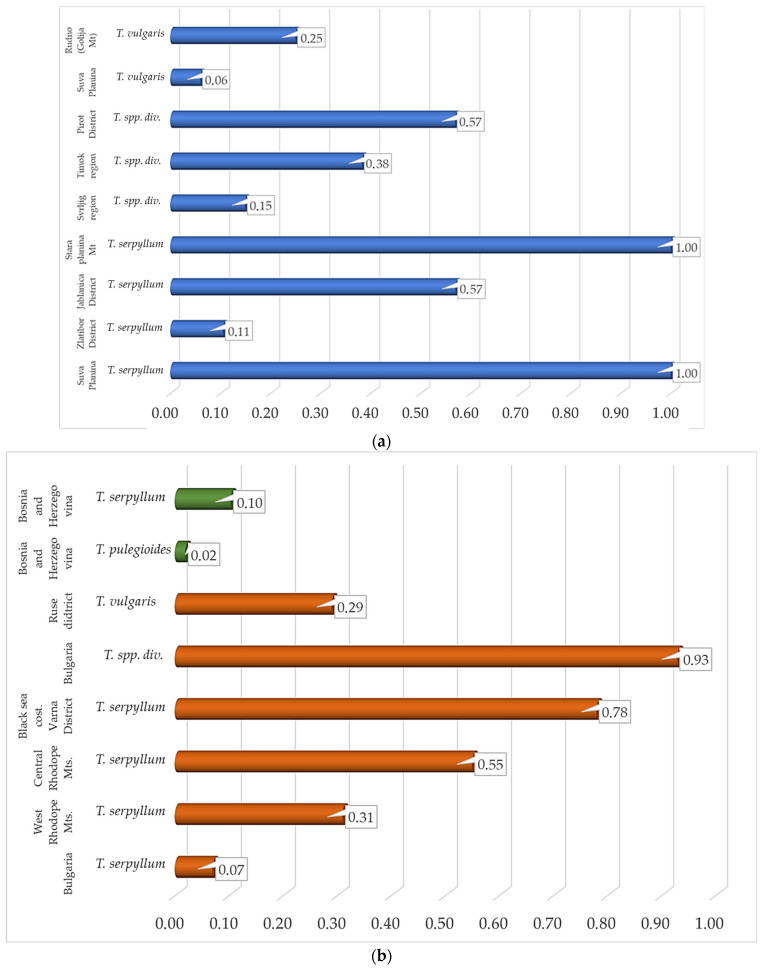

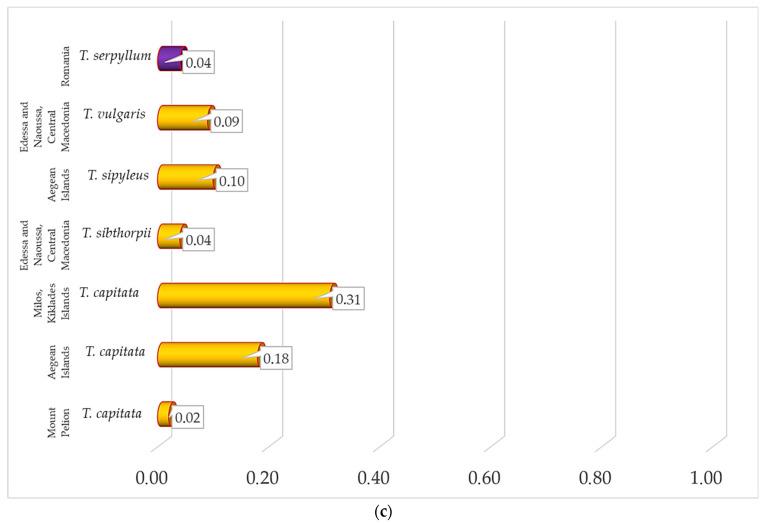
UV index across regions and *Thymus* species. (**a**) Serbia, (**b**) Bosnia and Herzegovina, (**c**) Romania and Greece.

In Bosnia and Herzegovina, traditional medicinal use of thyme is documented 127 times across eight publications Table 1, Table 2 and Table S1), including studies by Redžić et al. [44,53], Redžić [54], Šarić Kundalić et al. [31,55,56], Ginko et al. [57], and Muratović and Parić [58]. *T. aff. comosus*, *T. longedentatus*, *T. praecox*, and *T. pulegioides* are reported as remedies for a wide range of disorders, including liver ailments, sedative purposes, “blood purification”, bronchitis, asthma, diarrhea, flatulence, and stomach spasms [55]. Additional uses include treatment of cough, respiratory inflammations and infections, halitosis, diarrhea, childhood enuresis, insomnia, hangover, greasy skin, and for strengthening the body, as well as use as a roborant [31]. *T. serpyllum* is reported for the treatment of neurosis, cough, and stomach disorders [53], as well as for cough, pulmonary ailments, diarrhea, and liver and gallbladder disorders [57]. Additional applications include use as an analeptic, antianemic, anti-infective, and antipyretic agent, as well as diuretic, laxative, and hepatic remedies, and for the treatment of asthma, dysmenorrhea, gastritis, and hypertension [58]. *T. vulgaris* is reported for the treatment of dry cough and pulmonary ailments [57]. Records referring to *Thymus* sp. indicate its use for respiratory diseases, while *T. serpyllum* is also mentioned as a tranquilizer [44]. In southern Bosnia and Herzegovina, *T. pulegioides* is reported as a remedy for hysteria, epilepsy, and psychotic states of mind, whereas *T. serpyllum* is used to strengthen the heart muscle, treat heart diseases, act as a bronchial dilator, regulate menstruation, alleviate hand tremor, and for the “treatment of any sickness” [54]. Despite this wide range of medicinal applications, thyme is not among the most popular medicinal plants in the region, with relatively low use values (UVaverage = 0.06; UVmax = 0.10; Figure 2). Notably, no culinary use of thyme has been documented in Bosnia and Herzegovina.

#### 3.2.4. Bulgaria

In Bulgaria, traditional medicinal use of thyme is documented 33 times across 12 publications (Table 1, Table 2 and Table S1), including studies by Davidov et al. [59], Ploetz [60], Kalchev [61], Leporatti and Ivancheva [36], de Boer [62], Bertsch [63], Kozuharova et al. [64], Cherneva [65], Ivanova et al. [66], Mincheva et al. [35,67], and Boneva et al. [68]. Here, thyme is a relatively popular medicinal plant, particularly in certain regions, with an average use value (UVaverage) of 0.49 and a maximum use value (UVmax) of 0.93 (Figure 2). Various *Thymus* species, most commonly reported under the name *T. serpyllum*, are used primarily for the treatment of respiratory and gastrointestinal disorders; however, applications for disorders of the nervous system are reported with nearly equal frequency (Table 1, Table 2 and Table S1). In Bulgaria, thyme is known under a wide variety of local vernacular names, reflecting its cultural importance. These include mashterka, shterka, mashterica, mashteriga, matrina dusha, matorina dushitza, babina dushica, majchina dushica, yabulkinja, tchubritza, mashterka bela, materka, mahra, yabulchinja, volenika, volenica, ovcharska merudija, and ovcharska chubrica. In addition to its medicinal applications, thyme is widely used as a culinary seasoning, most commonly in dried and powdered form [59,60,66].

#### 3.2.5. Croatia

In Croatia, traditional medicinal use of thyme is documented 39 times across five publications, predominantly in relation to the treatment of respiratory diseases (Table 1, Table 2 and Table S1) [40,41,44,69,70]. In this context, *T. serpyllum* and *T. vulgaris* are traditionally used for a wide range of ailments, including respiratory conditions (e.g., bronchitis and asthma), gastrointestinal disorders (diarrhea, flatulence, and gastric ulcer), women’s diseases, kidney and bladder disorders, rheumatism, joint pain, and disorders of the nervous system, such as insomnia and neurasthenia [69]. On the Adriatic islands, *Thymus* spp., mainly *T. longicaulis* and *T. vulgaris*, are used to treat respiratory disorders, particularly the common cold [70]. In the Knin area (inland Dalmatia), *T. longicaulis* is administered as an infusion to treat cough, stomach ailments, fever in children, lung-related conditions, and the common cold [41]. In Istria, *Thymus* spp., predominantly *T. longicaulis*, are used as antitussive and sedative remedies, as well as for the treatment of skin conditions [40]. In northwestern Slavonia (continental Croatia), *T. serpyllum* is additionally used as a condiment, as a tea, and in the preparation of liqueurs [40]. Furthermore, *T. pulegioides* is reported to be used as a food seasoning, primarily in dried and powdered form [44].

#### 3.2.6. Greece

In Greece, traditional medicinal use of thyme is documented 21 times across three publications (Table 1, Table 2 and Table S1) [71,72,73,74]. Various *Thymus* species are used mainly for the treatment of respiratory and gastrointestinal disorders. Overall, thyme is not among the most popular medicinal plants in the country, showing relatively low use values (UVaverage = 0.12; UVmax = 0.31; Figure 2). In Central Macedonia, decoctions of T. sibthorpii are used to treat abdominal pain and gastrointestinal disturbances, whereas a decoction of *T. vulgaris* combined with flowers of *Sambucus nigra*, leaves of *Alcea rosea*, seeds of *Linum usitatissimum*, and aerial parts of *Verbascum longifolium* is employed as an expectorant, against cough, and for inflammations of the respiratory tract [72].

#### 3.2.7. Kosovo

In Kosovo, traditional medicinal use of thyme is documented 46 times across six publications, with applications predominantly related to the treatment of respiratory diseases, followed by gastrointestinal and nervous system disorders (Table 1, Table 2 and Table S1) [47,52,73,74,75,76]. In this context, *T. serpyllum* is widely used for the treatment of respiratory disorders, including bronchitis, asthma, and dry cough, as well as for gastrointestinal complaints such as diarrhea and as a carminative. Additional applications include use for sedation, treatment of skin burns, improvement of blood circulation, and as an anticholesterolemic, antidiabetic, and general health-promoting agent [73,74,75]. Furthermore, *T. longicaulis* is reported to be used as a mucolytic and digestive remedy, while *Thymus* spp. are employed as expectorants in cases of respiratory and lung inflammations and bronchitis [73].

#### 3.2.8. Montenegro

In this country, ethnobotanical records concerning the traditional medicinal use of thyme are notably scarce, with only a single publication documenting three medicinal applications (Table 1, Table 2 and Table S1) [77]. This limited number of records suggests that thyme does not occupy a prominent position in the local ethnomedicinal repertoire, at least in comparison with other Balkan regions where its use is more extensively documented. According to the available data, *T. serpyllum* is employed primarily for the treatment of gastrointestinal disorders and respiratory conditions, particularly spasmodic cough [77]. The narrow therapeutic scope and low frequency of reported uses may reflect regional preferences for other medicinal taxa, gaps in ethnobotanical documentation, or a decline in the transmission of traditional knowledge. Nevertheless, even this limited evidence confirms the inclusion of thyme within local traditional medicine and highlights its role as a remedy for common digestive and respiratory ailments.

#### 3.2.9. North Macedonia

In North Macedonia, traditional medicinal use of thyme is documented 21 times across six publications, with applications predominantly related to the treatment of respiratory diseases (Table 1, Table 2 and Table S1) [37,46,47,48,78,79]. In this context, *T. serpyllum* is reported to be used against respiratory disorders, including fever, influenza, and the common cold [46]. According to the same authors, *T. striatus* is employed in the treatment of dermatological conditions, as well as for reducing edema and promoting the elimination of excess body fluids, indicating its use in managing disorders related to fluid retention [46]

#### 3.2.10. Romania

In the southern part of Romania, which lies within the Balkan Peninsula, traditional medicinal use of thyme is documented eight times across four publications, with applications mainly related to the treatment of respiratory diseases (Table 1, Table 2 and Table S1) [42,80,81,82]. In this context, *T. serpyllum* is reported to be used primarily as an expectorant, as well as for its carminative and anti-infective properties. In addition, *T. vulgaris* is employed as a digestive and relaxant agent, used to alleviate cramps and to treat cough, while other species of the genus *Thymus* are also mentioned for similar therapeutic purposes (Table 1 and Table S1, Supplementary Material).

#### 3.2.11. Serbia

In Serbia, the number of recorded traditional medicinal uses of thyme reaches 164, representing the highest value on the Balkan Peninsula. These uses are documented across 20 publications and are primarily associated with the treatment of respiratory diseases, followed by disorders of the gastrointestinal and nervous systems (Table 1, Table 2 and Table S1) [39,83,84,85,86,87,88,89,90,91,92,93,94,95,96,97,98,99,100,101]. Within this broad ethnomedicinal framework, *T. glabrescens* is reported to be used for immune system strengthening, disease prevention, and the treatment of respiratory and digestive disorders [100]. *T. praecox* subsp. *jankae* is mentioned as a stimulant, sedative, and remedy against the common cold [93], while *T. pannonicus* is applied to treat nervous complaints, respiratory ailments, stomachache, and eye inflammations [90]. The use of *T. serpyllum* is particularly widespread and is documented both in classical folk medicine literature [83,84,85,86,87] and in numerous ethnobotanical studies [39,88,89,91,92,98,99]. Beyond medicinal applications, dried flowers of *T. serpyllum* are used as a spice, as well as for washing glassware and protecting pickled vegetables [39,84]. *T. vulgaris* is employed for the treatment of various ailments, particularly respiratory [98] and digestive disorders [94], and is additionally used as a culinary ingredient in honey, oils, bread, and pastries [85,86,102]. Furthermore, essential oils obtained from *T. glabrescens*, *T. malyi*, *T. marchallianus*, *T. pannonicus*, *T. praecox* subsp. *jankae*, *T. pulegioides*, and *T. striatus* are considered potential raw materials for the cosmetic and perfume industry [86]. In the Pirot District, several *Thymus* taxa were recorded, including *T. longicaulis*, *T. praecox* subsp. *jankae*, *T. praecox* subsp. *polytrichus*, *T. pulegioides* subsp. *pannonicus*, *T. pulegioides* subsp. *pulegioides*, *T. odoratissimus*, and *T. striatus* [95]. Notably, respondents generally do not distinguish between these taxa morphologically or in terms of use, referring to them collectively by the same vernacular names, such as babina dušica, dušičina, majčina dušica, or majkina dušica.

#### 3.2.12. Slovenia

In Slovenia, traditional medicinal use of thyme is documented only five times across two publications, indicating a relatively limited ethnomedicinal record compared to other Balkan countries. Reported applications are mainly related to disorders of the respiratory and reproductive systems (Table 1, Table 2 and Table S1) [38,103]. In this context, *T. serpyllum* is reported to be used for cardiovascular support, respiratory disorders, and the treatment of colds, as well as for gynecological problems such as menstrual cramps and to support breastfeeding mothers [38]. According to the same authors, various species of the genus *Thymus*, known under vernacular names such as poljska materina dušica, babja dušica, divji timijan, dušje, mačešica, materinka, preprišč, prežilka, and bukvica, are widely used in culinary contexts. These uses include application as spices, maceration in schnapps and herbal liqueurs, preparation of beverages and teas, and incorporation into soups, garnishes, egg, meat, fruit, and vegetable dishes, as well as desserts, highlighting the stronger culinary than medicinal role of thyme in Slovenian traditional practices.

#### 3.2.13. Turkey

In the European part of Turkey, traditional medicinal use of thyme is documented 49 times across five publications [104,105,106,107,108]. In contrast to most other Balkan countries, reported applications here are focused primarily on the treatment of gastrointestinal disorders and, to a lesser extent, respiratory ailments (Table 1, Table 2 and Table S1). Another distinguishing feature of this region is the taxonomic profile of the species used, with *T. longicaulis*, *T. sibthorpii*, and *T. zygioides* being the most frequently reported taxa, rather than *T. serpyllum* or *T. vulgaris* commonly cited elsewhere in the Balkans. Moreover, several therapeutic applications recorded in the European part of Turkey are not documented in other Balkan regions, including the use of thyme to treat nephritis and prostate-related conditions, as well as its application as an anti-dandruff and analeptic agent (Table 2 and Table S1, Supplementary Material).

#### 3.2.14. Quantitative Evaluation of *Thymus* Species Used Traditionally in the Balkans

Quantitative ethnobotanical indices, such as Use Value (UV), Quotation Frequency (QI) based on the number of spontaneous mentions, and Relative Frequency of Citation (RFC), are reported in only a limited number of the reviewed ethnobotanical studies (Table S2). The calculated UV values vary considerably among regions and among *Thymus* species (Figure 2), reflecting differences in the cultural relevance and intensity of use of thyme across the Balkan Peninsula. The UV index expresses the relative importance of a plant species within a given informant community, based on the frequency and diversity of its reported uses. In certain regions, thyme ranks among the most culturally significant medicinal plants, reaching a maximum UV value of 1, which indicates that the species is known and cited by all interviewed informants.

From a biological perspective, the dominance of respiratory and gastrointestinal applications across Balkan ethnobotanical records suggests a strong convergence between traditional therapeutic priorities and the pharmacological profile of *Thymus* species. The prevalence of antimicrobial, spasmolytic, and anti-inflammatory indications reflects the known activity spectrum of thymol- and carvacrol-rich essential oils, which are particularly relevant for respiratory tract conditions. Similarly, frequent references to digestive disorders may be associated with the carminative and antioxidant properties of phenolic compounds reported in several *Thymus* taxa. Although ethnobotanical data cannot be directly translated into clinical evidence, these patterns provide a biologically meaningful framework that supports further pharmacological and translational research.

### 3.3. Modes of Preparation and Administration

Overall, infusions (herbal teas) represent the most widely cited form of traditional administration of thyme. Common preparations involve dried aerial parts of the plant steeped in hot water (infusion) or boiled for a longer period (decoction). In Bulgaria and North Macedonia, for example, a typical preparation consists of 15 g of thyme prepared as an infusion or decoction in 500 g of water and consumed as a warm tea [45]. In addition to oral use, thyme preparations are also administered by inhalation, particularly for respiratory conditions [45,71,91].

The use of essential oils, applied either orally or externally, is frequently reported in Croatia, Greece, and Serbia. These oils are commonly mixed with honey, alcoholic beverages (e.g., rakija), or olive oil, depending on the intended therapeutic purpose. External applications are also well documented and include the use of baths [87], as well as alcoholic extracts prepared for massage or rubbing to relieve rheumatic pain and sprains [86]. Poultices are traditionally applied to purulent wounds, boils, effusions, burn wounds, contusions, and sprains [59]. Additional topical forms include compresses, ointments, and massage oils, particularly for the treatment of rheumatic pain, skin infections, and wound healing. Bath preparations containing thyme are reported from Slovenia and Montenegro, reflecting earlier historical records of its use as a sedative and tonic agent [12,13].

### 3.4. Cross-Cultural Patterns

In the Balkan Peninsula, delineating cultural and ethnobotanical boundaries strictly along modern political borders is often challenging. Several ethnic groups, such as the Gorani and Albanian communities, inhabit territories that extend across national borders [46,47,78]. As a result, traditional knowledge systems frequently transcend national boundaries, complicating attempts to analyze ethnobotanical patterns with high geographical precision. Despite this complexity, a clear regional pattern emerges, characterized by a strong overlap in medicinal practices across Balkan countries, reflecting shared historical, cultural, and ecological backgrounds. The use of thyme for respiratory ailments is nearly ubiquitous throughout the region, whereas its application for gastrointestinal disorders appears particularly emphasized in Bulgaria, Serbia, and Greece. In several countries, including Turkey, Bulgaria, and Albania, medicinal and culinary uses of thyme are closely intertwined, with the plant commonly employed both as a spice and as a health-promoting herbal infusion. The abundance and consistency of vernacular names for *Thymus* species, such as mashterka, babina dušica, majčina dušica, ovcharska merudija, and serpih, further illustrate the deep cultural integration of thyme into traditional healing practices and everyday life across the Balkans [36,59].

Knowledge of medicinal plants represents an important component of intangible cultural heritage that requires active preservation and transmission across generations. Its dissemination supports cultural identity, promotes sustainable development, and contributes to biodiversity conservation. Maintaining this continuity depends on effective intergenerational exchange of traditional ecological knowledge. In the contemporary digital era, engaging younger generations presents both challenges and opportunities, particularly as digitally mediated environments reshape learning practices. Within this context, media pedagogy and the development of media literacy offer promising tools for bridging traditional ethnobotanical knowledge with digitally oriented educational approaches [109,110].

### 3.5. Comparison with Modern Phytotherapy

The ethnobotanical evidence documented in this review shows strong concordance with modern pharmacological evaluations, including those summarized in the German Commission E and WHO monographs, which confirm the therapeutic efficacy of thyme preparations, particularly in the treatment of respiratory and gastrointestinal disorders [21,23]. For example, monoterpenoid phenols such as thymol and carvacrol are widely associated with antimicrobial, expectorant, and anti-inflammatory effects, which correspond well with the predominant traditional use of thyme for respiratory disorders. Likewise, phenolic compounds including rosmarinic acid have been linked to antioxidant and anti-inflammatory activities that may support the reported applications for gastrointestinal and immune-related conditions. These examples illustrate how selected phytochemical–pharmacological correlations help contextualize traditional knowledge within modern phytotherapeutic frameworks. Overall, traditional applications related to respiratory and gastrointestinal disorders appear to be the most strongly supported by modern pharmacological investigations, particularly due to the antimicrobial, expectorant, and spasmolytic properties associated with major constituents of *Thymus* essential oils. In contrast, several ethnomedical indications, including complex neurological conditions, cardiovascular disorders, or generalized “panacea”-type uses, remain only partially supported or lack sufficient clinical validation. These discrepancies do not diminish the ethnobotanical significance of such records but rather highlight important directions for future pharmacological and clinical research. The observed ethnobotanical patterns may also reflect differences in phytochemical profiles among *Thymus* taxa. Species rich in thymol and carvacrol are frequently associated with respiratory applications, which is consistent with their well-documented antimicrobial, expectorant, and anti-inflammatory properties. In contrast, taxa characterized by higher proportions of linalool and other oxygenated monoterpenes are often linked to calming, sedative, or nervine uses reported in traditional medicine, suggesting a possible biochemical basis for the prominence of nervous system-related applications. Although such correlations remain interpretative and require further targeted pharmacological studies, they provide a useful framework for understanding how chemical diversity may shape regional patterns of traditional use. This agreement between traditional use and regulatory assessment supports the long-standing medicinal relevance of *Thymus* species. Phytochemical investigations further substantiate these traditional applications. Rosmarinic acid has been identified as one of the predominant phenolic constituents across several *Thymus* species, with notably high concentrations reported in *T. pulegioides* (15,783.8 μg/g), followed by *T. callieri* (12,444.8 μg/g), *T. glabrescens* (11,667.4 μg/g), and *T. sibthorpii* (11,483.8 μg/g), whereas lower levels were observed in *T. zygioides* (7077.6 μg/g) [27]. Polyphenolic compounds, and rosmarinic acid in particular, are well known for their antioxidant and neuroprotective properties and have been associated with cognitive enhancement and memory improvement in experimental studies [111].

A clearer distinction can be made between traditional uses that are well supported by modern pharmacological and clinical evidence and those that remain insufficiently validated. The strongest concordance is observed for respiratory and gastrointestinal applications. The expectorant, antimicrobial, and spasmolytic activities of thymol- and carvacrol-rich preparations are well documented in pharmacological studies and acknowledged in Commission E and WHO monographs, thereby substantiating the traditional use of thyme in bronchitis, cough, and dyspeptic disorders.

In contrast, several reported applications, such as treatment of hypertension, anemia, epilepsy, prostate disorders, diabetes, or the traditional concepts of “blood purification” and panacea, lack sufficient clinical validation. Although some of these effects may be pharmacologically plausible based on antioxidant or anti-inflammatory properties, robust clinical evidence remains limited or absent.

Nervous system indications, including sedative and anxiolytic uses, occupy an intermediate position: experimental data suggest calming and neuroprotective effects, particularly in linalool- and rosmarinic acid-rich taxa, yet high-quality clinical trials are still scarce.

This distinction enhances the translational clarity of the ethnopharmacological data and identifies priority areas for future pharmacological and clinical research.

The most common chemotypes in the genus *Thymus* are defined by the dominance of specific monoterpene constituents—such as thymol, carvacrol, linalool, geraniol, and α-terpineol—and their derivatives in the essential oil profiles of many species. Extensive chemotypic diversity has been documented, with thymol and carvacrol often representing the principal phenolic monoterpenoids, and linalool, geraniol, and terpineol characterizing other chemotype classes across *Thymus* taxa. Thymol and carvacrol are among the most prevalent and quantitatively significant compounds identified in numerous *Thymus* essential oils, sometimes constituting 20–40% or more of the total volatile fraction in phenolic chemotypes [27,112,113,114,115]. From a phytochemical perspective, thymol and carvacrol are distinguished from many other monoterpenes by the presence of a phenolic hydroxyl group. This functional group confers higher chemical reactivity and is widely linked to pronounced antimicrobial, antifungal, and antioxidant activities observed in *Thymus* essential oils, distinguishing them from non-phenolic monoterpenes found in other chemotypes [4,27,114,115]. Recent experimental studies further support the strong antifungal potential of thyme essential oils; for example, essential oils from several Turkish Thymus species have demonstrated pronounced inhibitory activity against the phytopathogenic fungus Sclerotinia sclerotiorum, highlighting the broad antimicrobial potential associated with phenolic chemotypes [116].

The consistency between traditional knowledge and contemporary phytochemical and pharmacological findings underscores the relevance of *Thymus* species as valuable phytotherapeutic agents. Nevertheless, traditional preparations often lack standardization in dosage, composition, and extraction procedures, which limits their direct translation into evidence-based medical practice. In this context, regulatory evaluations play a crucial role. The Committee on Herbal Medicinal Products (HMPC) of the European Medicines Agency concludes that thyme preparations are indicated for the relief of productive (chesty) cough associated with the common cold. According to the European Medicines Agency monograph on Thymi herba, thyme-based herbal medicinal products are traditionally used as expectorants for productive cough associated with the common cold, reflecting long-standing medicinal use in European phytotherapy [117]. Importantly, these conclusions apply exclusively to specific, standardized preparations obtained from dried and cut leaves and flowers, expressed juice, or dry, liquid, and soft extracts produced using defined solvents (e.g., ethanol), followed by partial or complete solvent removal [118]. Such regulatory frameworks highlight the necessity of bridging traditional practices with standardized pharmaceutical formulations to ensure safety, efficacy, and reproducibility.

Implications for further research include identifying which traditional uses of *Thymus* taxa reported across Balkan countries are supported by evidence from modern pharmacological and clinical studies, and which traditional applications have not yet been substantiated. Additionally future research should investigate the relationship between the phytochemical profiles of *Thymus* taxa and their documented traditional areas of use. In particular, emphasis should be placed on clarifying associations between specific chemical compositions and therapeutic applications related to the respiratory and nervous systems. Furthermore, it is essential to elucidate the pharmacological roles of the principal constituents—thymol, carvacrol, and linalool—in shaping the observed ethnomedicinal use patterns. Such studies would contribute to a more mechanistic understanding of how phytochemical variability influences therapeutic efficacy and traditional knowledge systems.

## 4. Conclusions

The ethnobotanical evidence synthesized in this study demonstrates that species of the genus *Thymus* occupy a central and enduring position within the traditional medical systems, culinary practices, and cultural heritage of the Balkan Peninsula. Across all surveyed countries, thyme is consistently associated with the treatment of respiratory and gastrointestinal disorders, complemented by external applications for skin conditions, wound healing, and musculoskeletal complaints. This cross-cultural consistency, despite pronounced regional and linguistic diversity, reflects a shared ethnomedical framework shaped by long-standing cultural interactions and common ecological conditions.

The Balkan Peninsula harbors exceptional taxonomic diversity within *Thymus*, including numerous endemic and regionally restricted taxa, which represent both a valuable reservoir of traditional knowledge and a significant methodological challenge. Frequent taxonomic ambiguities and the interchangeable use of vernacular names highlight the need for integrative approaches combining ethnobotany, taxonomy, phytochemistry, and pharmacology. The strong concordance between dominant traditional uses and modern phytotherapeutic evaluations—particularly for respiratory and digestive applications—supports the empirical relevance of many folk practices, while also underscoring the limitations imposed by the lack of standardized preparation methods and species-level differentiation.

From a broader scientific and translational perspective, this synthesis bridges ethnobotanical knowledge with contemporary life sciences by identifying cross-cultural therapeutic patterns that may guide hypothesis-driven pharmacological research. The findings suggest that thyme-based preparations may continue to play a role in evidence-informed phytotherapy and public health strategies, provided that issues related to taxonomic accuracy, quality control, and safety assessment are addressed. Future research should prioritize species-resolved phytochemical and biological studies, comparative evaluations across Balkan regions, and clinical validation of key traditional indications. Such interdisciplinary efforts will contribute to the preservation, scientific interpretation, and sustainable application of *Thymus* species as culturally embedded yet pharmacologically relevant herbal resources.

## Figures and Tables

**Table 1:** Number of records in ethnobotanical publications documenting the traditional uses of thyme for various health conditions across Balkan countries. Legend: AL—Albania; BA—Bosnia and Herzegovina; BG—Bulgaria; HR—Croatia; GR—Greece; XK—Kosovo; ME—Montenegro; MK—North Macedonia; RO—Romania; RS—Serbia; SL—Slovenia; TR—Turkey. Health condition categories follow ethnomedicinal classification used in the cited sources.

	AL	BA	BG	HR	GR	XK	ME	MK	RO	RS	SL	TR	Total
Respiratory system	21	45	8	18	10	19	2	12	3	55	2	10	205
Gastrointestinal system	4	32	8	6	8	7	1	1	2	34		17	120
Nervous system	5	12	7	7		8		2	1	22		3	67
Urinary system		1		1		1		1		4		6	23
Cardiovascular system	1	7		1		2				5	1	2	19
Immune/lymphatic system	1	4	2	1		2		1		7			18
Integumentary system	1	5	1	1	1	3		3		2		1	18
Endocrine system and metabolism	1				1	3				4		5	14
Reproductive system		2	2	1						4	2	2	13
Skeletal & Muscular system			2	3					1	5			11
Other applications	3	1	3		1	1		1	1	22		3	45
Total	37	127	33	39	21	46	3	21	8	164	5	49	553

**Table 2:** Traditional application of *Thymus* sp. div. on the Balkan Peninsula—number of publications reporting the respective application. Legend: AL—Albania; BA—Bosnia and Herzegovina; BG—Bulgaria; HR—Croatia; GR—Greece; XK—Kosovo; ME—Montenegro; MK—North Macedonia; RO—Romania; RS—Serbia; SL—Slovenia; TR—Turkey, * taken internally, or baths.

Health Conditions	*Thymus* sp. div.	AL	BA	BG	HR	GR	XK	ME	MK	RO	RS	SL	TR
Respiratory system													
Angina	*T. vulgaris*				1								
Anti-tussive	*T. longicaulis*				1								
Anti-tussive	*T. pulegioides*						1						
Anti-tussive	*T. vulgaris*	1											
Asthma	* T. aff. comosus*		2										
Asthma	*T. longedentatus*		2										
Asthma	*T. longicaulis*												1
Asthma	*T. praecox.*		2										
Asthma	*T. pulegioides*		2										
Asthma	*T. serpyllum*	2	1				2						
Asthma	*T.* sp. div.										2		
Asthma	*T. vulgaris*				1								
Bronchial spasms	*T. serpyllum*										2		
Bronchial spasms	*T. vulgaris*										1		
Bronchitis	*T. aff. comosus*		1										
Bronchitis	*T. glabrescens*										1		
Bronchitis	*T. longedentatus*		1										
Bronchitis	*T. longicaulis*												1
Bronchitis	*T. praecox*		1										
Bronchitis	*T. pulegioides*		1										
Bronchitis	*T. serpyllum*	2	1				2		1		3		
Bronchitis	*T.* sp. div.						1				4		
Bronchitis	*T. vulgaris*				1								
Chills	* T. glabrescens*										1		
Chills	*T.* sp. div.										1		
Cold	* T. capitatus*					1							
Cold	* T. longicaulis*	1			2								3
Cold	* T. praecox*										1		
Cold	* T. pulegioides*										1		
Cold	* T. serpyllum*	2		1					2		2	1	
Cold	* T. sipyleus*					1							
Cold	* T. vulgaris*			1	1								
Cold	*Thymus* sp. div.	1			1						2		
Cough	*T. aff. comosus*		1										
Cough	* T. capitatus*	1				1							
Cough	* T. longedentatus*		1										
Cough	* T. longicaulis*				1								3
Cough	* T. praecox*		1										
Cough	* T. pulegioides*		1								1		
Cough	* T. pulegioides*		2										
Cough	* T. serpyllum*	2	2		1				3		2		
Cough	* T. sipyleus*					1							
Cough	* T. vulgaris*				1	1				1			
Cough	*Thymus* sp. div.			4						1	2		
Diphtheria	* T. vulgaris*				1								
Dry cough	*T. aff. comosus*		1										
Dry cough	* T. glabrescens*										1		
Dry cough	* T. longedentatus*		1										
Dry cough	* T. praecox*		1										
Dry cough	* T. pulegioides*		2										
Dry cough	* T. serpyllum*		1				1						
Dry cough	* T. vulgaris*		1										
Expectorant	* T. serpyllum*										2		
Expectorant	* T. serpyllum*						1			1	1		
Expectorant	* T. sipyleus*					1							
Expectorant	*T.* sp. div.						2				1		
Expectorant	* T. vulgaris*					1	1						
Fever/Antipyretic	* T. longicaulis*				1								
Fever/Antipyretic	* T. pulegioides*										1		
Fever/Antipyretic	* T. serpyllum*	2	1						2				
Fever/Antipyretic	*T.* sp. div.										2		
Influenza	* T. aff. comosus*		1										
Influenza	* T. longedentatus*		1										
Influenza	* T. longicaulis*												2
Influenza	* T. praecox*		1										
Influenza	* T. pulegioides*		2										
Influenza	* T. serpyllum*	4					2		2				
Influenza	*T.* sp. div.										2		
Lungs/lung inflammation	* T. longicaulis*				1								
Mucolytic	* T. longicaulis*						1						
Pharyngitis	* T. glabrescens*										1		
Productive cough	* T. glabrescens*										1		
Productive cough	*Thymus* sp. div.										1		
Pulmonary ailments	* T. serpyllum*		1										
Pulmonary ailments	* T. sipyleus*					1							
Pulmonary ailments	* T. vulgaris*		1										
Respiratory disorders	* T. longicaulis*				1								
Respiratory disorders	* T. serpyllum*			1			1	1	1		3	1	
Respiratory disorders	* T. vulgaris*				1						1		
Respiratory disorders	*Thymus* sp. div.				1						3		
Respiratory infections	*T. serpyllum*										1		
Respiratory inflammations	*T. serpyllum*	2					3						
Respiratory inflammations	* T. vulgaris*					1							
Sore throat/inflammation	* T. aff. comosus*		2										
Sore throat/inflammation	* T. capitatus*	1				1							
Sore throat/inflammation	* T. longedentatus*		2										
Sore throat/inflammation	* T. praecox*		2										
Sore throat/inflammation	* T. pulegioides*		2										
Sore throat/inflammation	* T. serpyllum*						1						
Sore throat/throat inflammation	*Thymus* sp. div.										2		
Spasmodic cough	* T. serpyllum*							1					
Stimulating respiratory system	*Thymus* sp. div.			1					1				
Whooping and regular cough	* T. vulgaris*										1		
Whooping cough	* T. serpyllum*										2		
Whooping cough	* T. vulgaris*				1								
Whooping cough	*Thymus* sp. div.										3		
** Gastrointestinal system **													
Abdominal pain	* T. atticus*												1
Abdominal pain	* T. longicaulis*												2
Abdominal pain	* T. sibthorpii*					1							
Against exceed secretion of bile	* T. serpyllum*										1		
Against exceed secretion of bile	* T. vulgaris*				1								
Appetite stimulant	* T. longicaulis*												1
Appetite stimulant	* T. serpyllum*			1									
Appetite stimulant	* T. sibthorpii*												1
Appetite stimulant	* T. zygioides*												1
Appetite stimulant	*Thymus* sp. div.										1		
Bad breath	* T. aff. comosus*		1										
Bad breath	* T. longedentatus*		1										
Bad breath	* T. praecox*		1										
Bad breath	* T. pulegioides*		2										
Bad breath	* T. serpyllum*						1						
Carminative/flatulence	* T. aff. comosus*		1										
Carminative/flatulence	* T. longedentatus*		1										
Carminative/flatulence	* T. praecox*		1										
Carminative/flatulence	* T. pulegioides*		1										
Carminative/flatulence	* T. serpyllum*	1					2			1	1		
Carminative/flatulence	* T. vulgaris*				1								
Diarrhea	* T. aff. comosus*		2										
Diarrhea	* T. longedentatus*		2										
Diarrhea	* T. praecox*		2										
Diarrhea	* T. pulegioides*		3										
Diarrhea	* T. serpyllum*		1				1				3		
Diarrhea	* T. vulgaris*				1						2		
Diarrhea	*Thymus* sp. div.			1							1		
Digestive	* T. vulgaris*									1			
Enteritis	* T. longicaulis*												1
Gastritis	* T. serpyllum*		1										
Gastrointestinal disorders/ailments	* T. serpyllum*	1					2	1			5		
Gastrointestinal disorders/ailments	* T. sibthorpii*					1							
Gastrointestinal disorders/ailments	* T. vulgaris*										1		
Gastrointestinal disorders/ailments	*Thymus* sp. div.										3		
Hertburn	* T. serpyllum*										1		
Improves digestion	* T. glabrescens*						1				1		
Improves digestion	* T. longicaulis*												1
Improves digestion	* T. sibthorpii*												1
Improves digestion	* T. zygioides*												1
Improves stomach function	* T. longicaulis*												1
Improves stomach function	* T. serpyllum*										1		
Improves stomach function	* T. sibthorpii*												1
Improves stomach function	* T. zygioides*												1
Laxative	* T. capitatus*					1							
Laxative	* T. serpyllum*		1										
Laxative	* T. sipyleus*					1							
Liver diseases/ailments	* T. aff. comosus*		1										
Liver diseases/ailments	* T. longedentatus*		1										
Liver diseases/ailments	* T. serpyllum*		2										
Liver diseases/ailments	*Thymus* sp. div.										1		
Regulates the level of stomach acid	* T. serpyllum*										1		
Rinsing the oral cavity/toothache	* T. capitatus*					2							
Rinsing the oral cavity/toothache	*Thymus* sp. div.			1							2		
Stimulating digestive system	*Thymus* sp. div.			1					1				
Stomach cancer	*Thymus* sp. div.			2									
Stomach disorders	* T. longicaulis*				1								1
Stomach disorders	* T. pulegioides *		2										
Stomach disorders	* T. serpyllum*		1								1		
Stomach disorders	* T. vulgaris*				1								
Stomach disorders	*Thymus* sp. div.			1							2		
Stomach spasms/pain	* T. aff. comosus*		1										
Stomach spasms/pain	* T. longedentatus*		1										
Stomach spasms/pain	* T. praecox*		1										
Stomach spasms/pain	* T. pulegoides*		1										
Stomach spasms/pain	*Thymus* sp. div.			1									
Stomachache	* T. glabrescens*										1		
Stomachache	* T. pulegoides*										1		
Stomachache	* T. serpyllum*										2		
Stomachache	* T. sibthorpii*												1
Stomachache	* T. vulgaris*										1		
Stomachache	*Thymus* sp. div.										1		
Toothache	* T. longicaulis*												1
Toothache (fruits)	* T. sibthorpii*												1
Ulcer duodenal	* T. capitatus*					1							
Ulcer duodenal	* T. sipyleus*					1							
Ulcer gastric	* T. vulgaris*				1								
** Nervous system **													
Analeptic	* T. serpyllum*		1										
Analeptic	* T. zygioides*												1
Anxiety	* T. serpyllum*										2		
Anxiety	* T. vulgaris*										1		
Convulsions	* T. serpyllum*										1		
Dizziness	* T. vulgaris*				1								
Epilepsy	*Thymus* sp. div.										2		
Eye inflammations	* T. pulegoides*										1		
For nerves	*Thymus* sp. div.										2		
Hand tremor treatment	* T. serpyllum*		1										
Headache	* T. serpyllum*	1											
Headache	*Thymus* sp. div.			1							2		
Hysteria, epilepsy and psychotic state of mind treatment	* T. pulegoides*		1										
Improves mood	* T. serpyllum*										1		
Improving hearing	*Thymus* sp. div.										1		
Insomnia (filing the pilow)	* T. pulegoides*		1										
Insomnia (filing the pilow)	*Thymus* sp. div.										1		
Insomnia/sedation/sedative	* T. aff. comosus*		1										
Insomnia/sedation/sedative	* T. longedentatus*		1										
Insomnia/sedation/sedative	* T. longicaulis*				1								2
Insomnia/sedation/sedative	* T. praecox*		1								1		
Insomnia/sedation/sedative	* T. pulegoides*		1										
Insomnia/sedation/sedative	* T. serpyllum*	1	1		1		4				2		
Insomnia/sedation/sedative	* T. vulgaris*				1								
Insomnia/sedation/sedative	*Thymus* sp. div.										2		
Nervous troubles	* T. pulegioides*										1		
Nervousness due to alcoholism	* T. vulgaris*				1								
Neurasthenia	* T. serpyllum*										1		
Neurasthenia	* T. vulgaris*				1								
Neurasthenia	*Thymus* sp. div.			1									
Neurosis	* T. pulegoides*		2										
Neurosis	* T. serpyllum*		1										
Relaxant/calmative/tranquilizer	* T. serpyllum*	1		1			2				1		
Relaxant/calmative/tranquilizer	* T. vulgaris*									1			
Relaxant/calmative/tranquilizer	*Thymus* sp. div.			2					1				
Spasmolytic	* T. serpyllum*	1					2						
Spasmolytic and analgetic	*Thymus* sp. div.			1									
Stimulating effect on nervous systems	*Thymus* sp. div.			1					1				
Tremor	* T. vulgaris*				1								
** Urinary system **													
Bed wetting by children	* T. longedentatus*		1										
Bed wetting by children	* T. praecox*		2										
Bed wetting by children	* T. pulegoides*		1										
Bed wetting by children	* T. serpyllum*						1						
Bed wetting by children	* T. aff. comosus*		1										
Diuretic	* T. longicaulis*												1
Diuretic	* T. serpyllum*		1										
Inflammation of the urinary tract	* T. serpyllum*										1		
Kidney and bladder diseases	* T. sibthorpii*												1
Kidney and bladder diseases	* T. vulgaris*				1						2		
Kidney and bladder diseases	*Thymus* sp. div.								1		1		
Kidney and bladder diseases	*Thymus* sp. div.												
Kidney stones/Renal stones	* T. aff. comosus*		1										
Kidney stones/Renal stones	* T. longedentatus*		1										
Kidney stones/Renal stones	* T. longicaulis*												1
Kidney stones/Renal stones	* T. praecox*		1										
Kidney stones/Renal stones	* T. pulegoides*		1										
Kidney stones/Renal stones	* T. sibthorpii*												1
Nephritis	* T. longicaulis*												2
** Cardiovascular system **													
Anemia	* T. vulgaris*				1								
Blood purification	* T. aff. comosus*		1										
Blood purification	* T. longedentatus*		1										
Blood purification	* T. praecox*		1										
Blood purification	* T. pulegioides*		1										
For the heart/hearth diseases	* T. longicaulis*												2
For the heart/hearth diseases	* T. serpyllum*		1								1	1	
For the heart/hearth diseases	*Thymus* sp. div.										2		
High blood pressure/Hypertension	* T. serpyllum*		1										
High blood pressure/Hypertension	*Thymus* sp. div.										2		
To improve blood circulation	* T. serpyllum*	1					2						
To strengthen heart muscle	* T. serpyllum*		1										
** Immune/lymphaic system **													
As roborantium/stregnthening the corpus	* T. aff. comosus*		1										
As roborantium/stregnthening the corpus	* T. longedentatus*		1										
As roborantium/stregnthening the corpus	* T. praecox*		1										
As roborantium/stregnthening the corpus	* T. pulegoides*		1										
Disease prevention	* T. glabrescens*										1		
Disease prevention	*Thymus* sp. div.										2		
Improving the immune system/immunostimulant	* T. glabrescens*										1		
Improving the immune system/immunostimulant	* T. serpyllum*	1					2						
Improving the immune system/immunostimulant	*Thymus* sp. div.										2		
Lymph node inflammation	* T. serpyllum*			1									
Preventive use against infectious diseases	* T. serpyllum*										1		
Scrofula (tuberculous lymphadenitis)	* T. vulgaris*				1								
Scrofula (tuberculous lymphadenitis)	*Thymus* sp. div.			1					1				
** Integumentary **													
Anti-dandruff	* T. sibthorpii*												1
Dermatological system problems	* T. striatus.*								2				
Disinfecting skin/cosmetic preparations—antiseptic	* T. vulgaris*										1		
For greasy skin	* T. aff. comosus*		1										
For greasy skin	* T. longedentatus*		1										
For greasy skin	* T. praecox*		1										
For greasy skin	* T. pulegoides*		2										
For greasy skin	* T. serpyllum*						1						
Fungal diseases—topical treatment	* T. vulgaris*										1		
Minimize edema, remove fluid	* T. striatus.*	1					1		1				
Purulent wounds, boils, effusions, burn wounds, contusions, sprains	*Thymus* sp. div.			1									
Skin burns	* T. serpyllum*						1						
Skin treatment	* T. longicaulis*				1								
Wounds	* T. capitatus*					1							
** Endocrine system and metabolism **													
Anti-cholesterolemic	* T. longicaulis*												1
Anti-cholesterolemic	* T. serpyllum*						1						
Anti-cholesterolemic	* T. vulgaris*	1					1						
Antidiabetic/Diabetes mellitus	* T. longicaulis*												2
Antidiabetic/Diabetes mellitus	* T. zygioides*												2
Antidiabetic/Diabetes mellitus	*Thymus* sp. div.					1	1				2		
Thyroid diseases	*Thymus* sp. div.										2		
** Reproductive system **													
For breastfeeding mothers	* T. serpyllum*											1	
Gynecological complaints/Women’s diseases	* T. serpyllum*										1	1	
Gynecological complaints/Women’s diseases	* T. vulgaris*				1								
Menstrual pain/Dysmenorrhea	* T. serpyllum*		1										
Menstrual pain/Dysmenorrhea	*Thymus* sp. div.			2							2		
Menstrual problems/Menstrual cycle disorder	* T. glabrescens*										1		
Menstruation irregular/frequent/period regulation	* T. serpyllum*		1										
Prostate	* T. sibthorpii*												2
** Skeletal & Muscular **													
Cramps	* T. vulgaris*									1			
Gout—joint disease	* T. serpyllum*										1		
Joint dislocation	* T. vulgaris*				1								
Joint dislocation	*Thymus* sp. div.			1									
Joint inflammation *											1		
Joint pain	* T. vulgaris*				1								
Rachitis	*Thymus* sp. div.										1		
Rheumatic pains and sprains—alcoholic extracts for rubbing in	* T. serpyllum*										1		
Rheumatism	* T. vulgaris*				1								
Rheumatism	*Thymus* sp. div.			1							1		
** Others **													
Alcoholism/hangover	* T. aff. comosus*		1										
Alcoholism/hangover	* T. longedentatus*		1										
Alcoholism/hangover	* T. praecox*		1										
Alcoholism/hangover	* T. pulegoides*		1										
Alcoholism/hangover	* T. serpyllum*										1		
Alopecia	* T. capitatus*					1							
Anthelmintic/intestinal parasites	* T. longicaulis*												1
Anthelmintic/intestinal parasites	* T. serpyllum*										2		
Anthelmintic/intestinal parasites	*Thymus* sp. div.										2		
Antibiotic	* T. serpyllum*										1		
Anti-infective	* T. serpyllum*		1							1			
Anti-inflammatory/Inflammation	* T. longedentatus*		1										
Anti-inflammatory/Inflammation	* T. longicaulis*												1
Anti-inflammatory/Inflammation	* T. praecox*		1										
Anti-inflammatory/Inflammation	* T. pulegoides*		1										
Anti-inflammatory/Inflammation	*Thymus* sp. div.										2		
Antiseptic	* T. serpyllum*										5		
Antiseptic	* T. vulgaris*										1		
Antiseptic	*Thymus* sp. div.										3		
Antiviral	* T. serpyllum*										1		
Aromatic	* T. serpyllum*										1		
Foot-and-mouth disease in livestock (aphthae epizooticae, apthous fever)	*Thymus* sp. div.			1									
For rubbing													
Infectious diseases	* T. vulgaris *										1		
Inflammation	* T. aff. comosus*		1										
Loosing of weight	* T. longicaulis*												1
Medicinal (not specified)	*Thymus* sp. div.			2									
Panacea	* T. pulegoides*	1									1		
Panacea	* T. serpyllum*		1				1						
Panacea	* T. longicaulis*	2							1				
Stimulant	* T. praecox*										1		
Vermifuge													
** Culinary **													
Used as a food seasoning (dried and powdered)	* T. pulegioides*	2			1								
Used as a food seasoning (dried and powdered)	*Thymus* sp. div.			1								3	
Beverage preparing wine, flavouried with Arthemisia species and other herbs and/or fruits	*Thymus* sp. div.			1									
Maceration in schnapps	*Thymus* sp. div.											1	

## Data Availability

No new data were created or analyzed in this study.

## Supplementary Materials

The following supporting information can be downloaded at: https://www.mdpi.com/article/10.3390/life16030452/s1, Table S1: Traditional application of *Thymus* sp. div. on the Balkan Peninsula—number of publications reporting the respective application. Legend: AL—Albania; BA—Bosnia and Herzegovina; BG—Bulgaria; HR—Croatia; GR—Greece; XK—Kosovo; ME—Montenegro; MK—North Macedonia; RO—Romania; RS—Serbia; SL—Slovenia; TR—Turkey, * taken internally, or baths; Table S2: Ethnobotanical indices for *Thymus* species used in the Balkan countries.
